# Reliability of a 3 T MRI protocol for objective grading of supraspinatus tendonosis and partial thickness tears

**DOI:** 10.1186/s13018-014-0128-x

**Published:** 2014-12-18

**Authors:** Stefan Bauer, Allan Wang, Rodney Butler, Michael Fallon, Robert Nairn, Charley Budgeon, William Breidahl, Ming-Hao Zheng

**Affiliations:** Department of Orthopaedic Surgery, Sir Charles Gairdner University Hospital, Perth, PO Box 1440, 6904 Subiaco Australia; Perth Radiological Clinic, 127 Hamersly Road, 6009 Subiaco, Australia; Centre of Applied Statistics, The University of Western Australia, 35, Stirling Highway, 6009 Crawley, Western Australia; School of Surgery, Centre of Orthopaedic Research, The University of Western Australia, 35, Stirling Highway, 6009 Crawley, Western Australia

**Keywords:** Partial thickness supraspinatus tear, Tendonopathy, Tendonosis, MRI, Inter-observer, Intra-observer, Reliability

## Abstract

**Background:**

Partial thickness supraspinatus tears and tendonosis can be managed either nonoperatively or by various arthroscopic techniques. New biologic treatment approaches are currently being investigated. MRI is commonly used for objective imaging outcome evaluation but there is a lack of reliability studies. We propose a novel MRI classification of partial supraspinatus tears and tendonosis and evaluate its inter-observer and intra-observer reliability.

**Methods:**

Digital MRI scans (3 Tesla) of 65 patients investigated for assessment of supraspinatus pathology or subacromial impingement were evaluated by three independent and experienced musculoskeletal (MSK) radiologists. Tendonosis (absent, focal, generalized), partial thickness (PT) tears (absent, 0%–25% PT, 25%–50% PT, 50%–100% PT, and full thickness tears), and anteroposterior extent of tears (less than 5 mm, 5–10 mm, greater than 10 mm) were scored by each radiologist on two separate occasions (t1, t2), 2 months apart. The inter-observer and intra-observer agreement and weighted kappa values for each parameter were calculated.

**Results:**

The range of weighted intra-observer kappa (IAK) was 0.84–0.93 for evaluation of tendonosis; 0.84 (all raters) for depth of partial thickness, 0.74–0.84 for AP tear size, and 0.83–0.85 for the total score. The range of weighted inter-observer kappa (IEK) over two time points (t1, t2) was 0.55–0.74 for tendonosis, 0.69–0.84 for depth for partial thickness tears, 0.57–0.80 for AP tear size, and 0.63–0.80 for the total score.

**Conclusion:**

A comprehensive MRI grading protocol is proposed and is reliable for the evaluation of supraspinatus tendonosis and partial thickness tears with good to excellent kappa values. This rotator cuff MRI protocol can be applied to evaluate morphological tendon outcomes after different treatment modalities.

**Electronic supplementary material:**

The online version of this article (doi:10.1186/s13018-014-0128-x) contains supplementary material, which is available to authorized users.

## Introduction

Rotator cuff pathology varies from tendonosis and partial thickness tears, to full thickness tendon tears and in some cases, progression to rotator cuff arthropathy. MRI has been shown to be reliable in the evaluation of full thickness tendon tears [1-3]. The evaluation of the extent of full thickness tendon tears and tendon retraction has good intra-observer and inter-observer agreement when assessed by multiple observers [2,3].

Tendonosis also named tendinosis and partial rotator cuff tears are part of the broad spectrum of rotator cuff disease. Rotator cuff disease can be divided into rotator cuff tendonopathy (including external and internal impingement, tendonitis, tendinosis with degeneration, and partial thickness tendon tears) and full thickness tears [4]. Tendonosis and partial thickness cuff injuries are prevalent in heavy sporting activity and in middle age [5]. They tend to increase with age, supporting the contribution of tendon degeneration as an important causative factor [4]. When symptomatic, many partial tears can be successfully managed with nonoperative treatment. Some may spontaneously heal, but this is rare and many partial tears will enlarge [6]. The management of more extensive partial cuff tears is complex, with treatment options including arthroscopic debridement [7,8], arthroscopic decompression [7,9], trans-tendon repair of the partial tear, take down and full thickness repair of the partial tear [10,11], and innovative biologic therapies such as platelet-rich plasma and stem cell injections [12-15] which require further investigations in high-quality studies to delineate their value. To evaluate the treatment outcome, or compare the efficacy of different treatments for partial thickness supraspinatus tears, an objective and reliable measure of supraspinatus tendon healing is required.

MRI is a readily available, non-invasive, and safe imaging modality. MRI has been used to grade cuff tendonopathy, and MRI reliability has been assessed between multiple observers. Sein et al. [16] scored tendonopathy with four categories (normal, mild focal, moderate focal, severe generalized) and reported good agreement with a single well-trained observer (intra-class correlation 0.85), but only fair to good inter-observer reliability (ICC 0.55).

Sugaya et al. [17] used five grades to evaluate rotator cuff healing after rotator cuff repair surgery. However, this system does not assess the severity of tendonosis independently of coexisting cuff tears. Partial thickness tendon tears are assessed qualitatively as ‘sufficient’ or ‘insufficient’. This evaluation protocol has not been evaluated for intra-observer or inter-observer reliability.

Castricini et al. [13] used a scoring system from 3–9 to evaluate tendonopathy, foot print coverage, and tendon thickness in an outcome study of arthroscopic rotator cuff repairs with platelet-rich plasma. Assessments were made by orthopedic surgeons with no reliability data available for this scoring protocol.

Spencer et al. [3] performed an inter-observer agreement study of 27 MRI scans by ten orthopedic surgeons. Assessment of partial cuff tendon damage included bursal or articular surface involvement (moderate agreement, kappa = 0.44) and less than or greater than 50% tear of cuff tendon thickness (poor agreement, kappa = −0.11). This study indicates that a high level of MRI expertise is likely to be required to make a comprehensive MRI assessment of the rotator cuff reliable. Along with previous studies, this study further highlights the broad spectrum of rotator cuff pathology that can be potentially evaluated by MRI. Tendonosis can exist alone as evaluated in the study by Sein at al. [16] or in combination with partial thickness tears of varying severity. Partial tendon tears occur in three dimensions involving variables of tear thickness, mediolateral retraction, and extension in the anteroposterior direction. These variables in rotator cuff tendonopathy are important to appreciate to enable selection of the optimal treatment and also comprehensive assessment of treatment outcome.

The objectives of this study are to introduce a new MRI scoring protocol for differentiated assessment of supraspinatus partial tears and tendonosis. Secondly, this study aims to evaluate the reliability of this MRI protocol by measuring intra-observer and inter-observer agreement among experienced musculoskeletal (MSK) radiologists. Our hypothesis is that this MRI protocol is reliable and can therefore be used for surveillance of partial thickness supraspinatus rotator cuff lesions or evaluation of the tendon response to treatment.

## Material and methods

### MRI scans and patients

MRI scans of patients referred for evaluation of impingement syndrome or supraspinatus tear were screened by an independent MSK radiologist who did not participate as an observer. This was undertaken after a formal MRI report was generated.

Inclusion criteria for the study cohort were scans demonstrating a normal supraspinatus tendon, supraspinatus tendonosis, and low- to high-grade partial thickness supraspinatus tears. Exclusion criteria were patient age younger than 18 years or older than 65 years and full thickness supraspinatus tears with any medial retraction.

The independent radiologist selected and included 65 shoulder MRI scans of patients out of a larger pool of scans and this selection was exclusively based on the study selection criteria as outlined above. There were 255 scans identified for the search criteria in the referral (supraspinatus tear or impingement syndrome) in the study period. Of those scans, 126 scans were excluded for patient age (>65 or <18 years) and a further 64 scans for full thickness tears. All scans were undertaken on a 3 Tesla MRI scanner in 2012/2013 (January 2012–August 2013) in one institution. Institutional review board approval was received from the Perth Radiological Clinic CMC Board prior to commencement of the study.

The patient demographics were as follows: 30 females (46%) and 35 males (54%) with a mean age of 45 years (range: 18–65 years). Scans were randomly numbered (1–65), de-identified of patient data, and blinded for the formal reports of the scans.

### MRI

The patients were scanned on a 3 Tesla Ingenia MRI scanner (Phillips, Eindhoven, The Netherlands). All studies were performed in a dedicated eight-channel shoulder coil.

Coronal PD (TR = 3,000 ms, TE = 25 ms) and T2w fat-suppressed (TR = 3,000 ms, TE = 60 ms) images were obtained with a 14-cm FOV, 3-mm slice thickness with a 0.3-mm slice gap. Sagittal PD (TR = 3,000 ms, TE = 25 ms) and T2w fat-suppressed (TE = 3,000 ms, TE = 80 ms) images were obtained with a 14-cm FOV, 3-mm slice thickness and 1-mm slice gap.

Axial PD fat-suppressed (TR = 2,800 ms, TE = 30 ms) images were obtained with a 14-cm FOV, 3.5-mm slice thickness and 1-mm slice gap.

### Study observers and scoring

Three fellowship-trained MSK radiologists with 18, 11, and 3 years of post-fellowship experience were test observers for the study. Each observer scored the 65 MRI scans independently on two occasions (t1 and t2), 2 months apart at a single location. The MSK radiologists underwent a training session at the onset of the study. The scoring system used to grade the supraspinatus partial thickness tendon injury was introduced. A meeting was held prior to the first scoring session (t1) and was repeated prior to the second scoring session 2-months later (t2). Radiologists were blinded as to the scores from the two other participating radiologists, from the scores from their own initial scoring session (t1), from all patient data, and from the formal reports of the MRI scans. All MRI scans were evaluated on high-resolution digital radiology monitors at a single center and using InteleViewer software.

An instructional scoring questionnaire (Additional file 1) and scoring template (Table 1) were completed for each scan.

**Table 1:** **MRI scoring template for supraspinatus tendonosis and partial thickness tears**

**Domain**	**Score = 0**	**Score = 1**	**Score = 2**	**Score = 3**	**Score = 4**
*Tendonosis*	**□** Normal tendon: uniform low signal on pd and t2 weighted images	**□** Focal tendonosis: increased signal on pd images or increased signal on t2 weighted images (*not fluid signal*) extending *not* more than 10 mm in any dimension	**□** Generalized tendonosis: increased signal on pd or t2 weighted images (*but not fluid signal*) extending more than 10 mm in any dimension	N/A	N/A
*Tear thickness*	**□** No tear	**□** < 25% tendon thickness	**□** 25% to < 50% tendon thickness	**□** 50% to <100% tendon thickness	**□** Full thickness
*AP tear size in mm*	**□** No tear	**□** < 5	**□** 5*–*10	**□** > 10	N/A
*Total 0–9*					

For tendonosis, recognized MRI features are increased signal intensity and/or swelling and increased tendon thickness [18]. Increased signal intensity has been histologically correlated with myxoid collagen degeneration, splits in the tendon, and increased type III collagen [19,20]. The signal intensity for tendonosis is lower than the intensity of fluid signal which represents disruption of collagen fibers and tendon tears.

**Figure 1:**
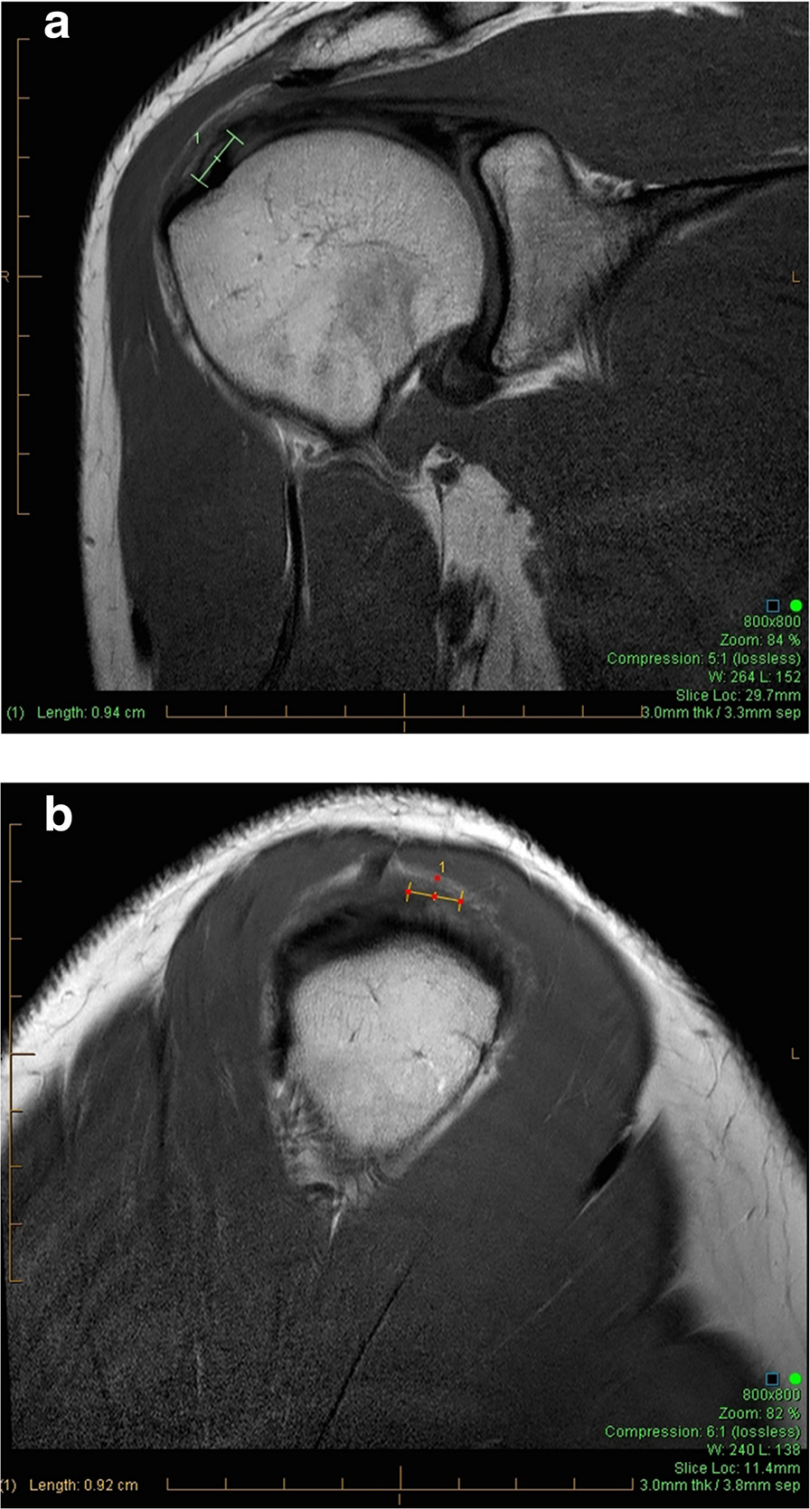
**Focal tendonosis 9 × 9 mm, coronal (a) and sagittal (b).**

Tendonosis is scored (0, 1, 2) on coronal and sagittal scans. The greatest extent of tendonosis was identified by scrolling from anterior to posterior on coronal images and from lateral to medial on sagittal images. The dimensions of tendonosis were measured using the InteleViewer software. A score of 0 is given for a normal tendon on PD and T2 images. A score of 1 indicates focal tendonosis where signal increase is non-fluid signal intensity and is not measured greater than 10 mm in maximal extent in any dimension (Figure 1). A score of 2 indicates generalized tendonosis where signal intensity is non-fluid signal intensity and is measured as greater than 10 mm in any dimension.

**Figure 2:**
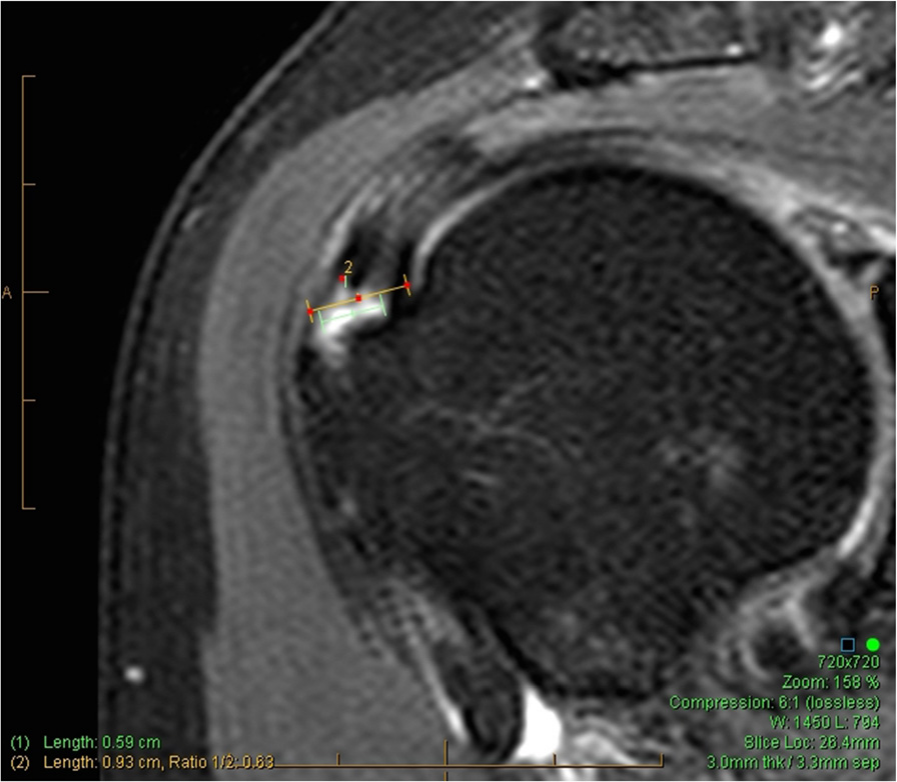
**63% partial tear.**

Tear thickness is scored 0–4 on coronal T2 weighted images while scrolling from anterior to posterior. A tendon tear is identified by fluid signal intensity. The highest grade tear thickness is identified. The total tendon thickness at that location is calculated by measuring the perpendicular distance from the greater tuberosity footprint to the bursal tendon surface. The thickness of the partial tendon tear is calculated as a percentage of the total tendon thickness at that location (Figure 2).

**Figure 3:**
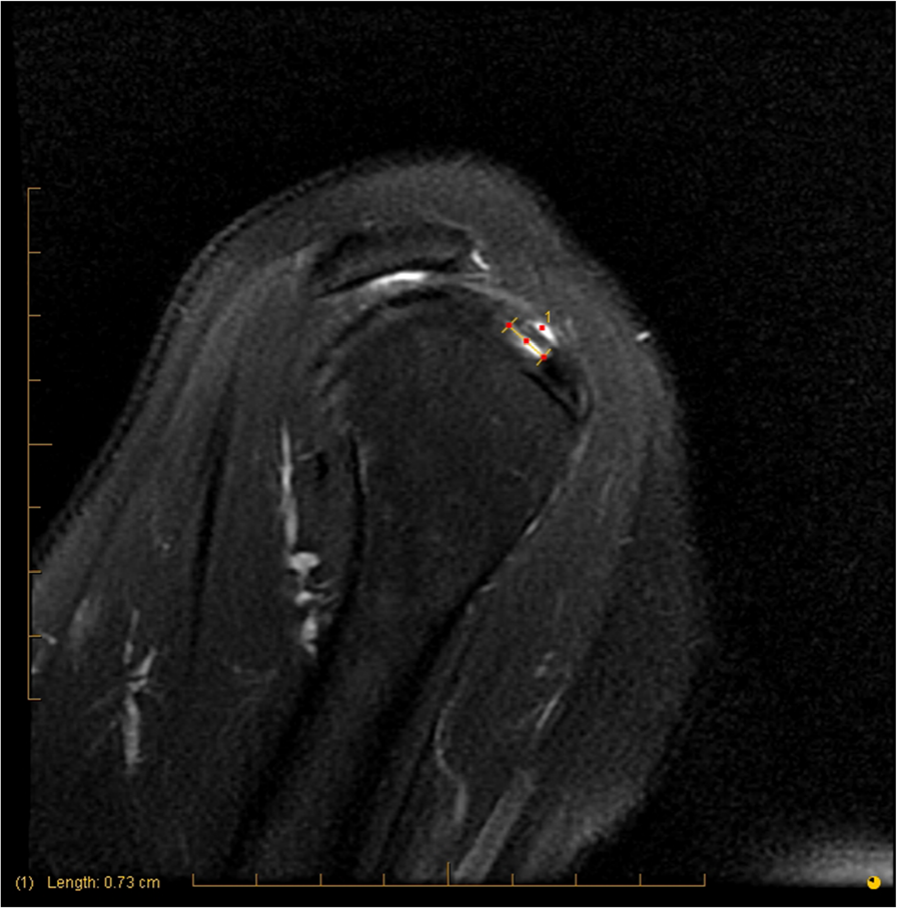
**Scoring: 7-mm AP tear.**

AP tear size is measured and scored 0–3 on sagittal T2 weighted images while scrolling from medial to lateral toward the supraspinatus footprint. The greatest extent of a tear is measured in the AP dimension (Figure 3).

A total tendonopathy score is calculated from the sum of tendonosis, tear thickness, and AP tear size scores. A tendonopathy total score will vary from 0 (normal tendon) to a maximum score of 9 (generalized tendonosis with a full thickness tear more than 10 mm in AP dimension).

### Statistical methods

Weighted kappa statistics were used to evaluate intra-observer and inter-observer agreement (intra- and inter-observer kappa: IAK and IEK). Kappa statistics measure agreement beyond the agreement due to chance alone (expected agreement). Weighted kappa statistics measure agreement between two observers for ordinal scoring scales taking into account how close their agreement is (e.g. 1 and 2 is a better agreement than 1 and 3). Weighted kappa values for the three raters, 95% confidence intervals (95% CI), standard error (SE), and *P*-values, were calculated. According to the guidelines by Landis and Koch [21], we classified agreement in relation to kappa values as being excellent (0.81 to 1), good (0.61 to <0.81), moderate (0.41 to <0.61), fair (0.20 to <0.41), poor (0 to <0.20), and less than chance (<0). The data was analyzed using the R environment for statistical computing (R foundation for statistical computing, Vienna, Austria, 2012).

## Results

### Reliability of supraspinatus partial thickness tear and tendonosis grading

#### Intra-observer reliability trial

Table 2 shows the weighted IAK’s of three observers for all score domains.

**Table 2:** **Average weighted intra-observer** kappa **(IAK, time points t1 and t2)**

**Comparisons**	**Percentage agreement**	**Weighted kappa**	**Standard error**	**Confidence interval**	***P*** **-value**
*Tendonosis* (*IAK; t1, t2*)					
Rater 1	93.9%	0.93	0.03	(0.86, 1.00)	<0.0001
Rater 2	90.8%	0.85	0.06	(0.74, 0.97)	<0.0001
Rater 3	87.7%	0.84	0.05	(0.74, 0.95)	<0.0001
*Range*	88%–94%	0.84–0.93			
*Mean*	90.8%	0.88			
*Tear thickness* (*IAK; t1, t2*)					
Rater 1	78.5%	0.84	0.04	(0.75, 0.92)	<0.0001
Rater 2	81.5%	0.84	0.04	(0.76, 0.93)	<0.0001
Rater 3	78.5%	0.84	0.04	(0.76, 0.92)	<0.0001
*Range*	79%–82%	0.84			
*Mean*	79.5%	0.84			
*AP extent* (*IAK; t1, t2*)					
Rater 1	72.3%	0.74	0.06	(0.63, 0.84)	<0.0001
Rater 2	78.5%	0.84	0.04	(0.76, 0.91)	<0.0001
Rater 3	80.0%	0.83	0.04	(0.74, 0.92)	<0.0001
*Range*	72%–80%	0.74–0.84			
*Mean*	76.9%	0.80			
*Total score* (*IAK; t1, t2*)					
Rater 1	61.5%	0.83	0.03	(0.76, 0.89)	<0.0001
Rater 2	63.1%	0.85	0.03	(0.79, 0.91)	<0.0001
Rater 3	58.5%	0.84	0.02	(0.79, 0.90)	<0.0001
*Range*	59%–63%	0.83–0.85			
*Mean*	61.0%	0.84			

The mean IAK for tendonosis was 0.88 (range: 0.84–0.93), for tear thickness 0.84 (range: 0.84 for all raters), for AP tear size 0.80 (range: 0.74–0.84), and for total tendonopathy score 0.84 (range: 0.83–0.85). According to Landis and Koch [21], these mean kappa values reflect excellent (0.81–1.00) and good agreement (0.61–0.80).

#### Inter-observer reliability trial

Table 3 shows the weighted IEK’s of three observers for all score domains at the two time points (t1, t2).

**Table 3:** **Average weighted inter-observer** kappa **(IEK, time point t1 and t2)**

**Comparison**	**Percentage agreement**	**Weighted kappa**	**Standard error**	**Confidence interval**	***P*** **-value**
*Tendonosis* (*IEK*)					
Rater 1, rater 2, t1	81.5%	0.74	0.07	(0.59, 0.88)	<0.0001
Rater 1, rater 3, t1	67.7%	0.55	0.09	(0.38, 0.72)	<0.0001
Rater 2, rater 3, t1	81.5%	0.72	0.08	(0.57, 0.87)	<0.0001
Rater 1, rater 2, t2	80.0%	0.73	0.07	(0.60, 0.87)	<0.0001
Rater 1, rater 3, t2	76.9%	0.65	0.08	(0.49, 0.82)	<0.0001
Rater 2, rater 3, t2	80.0%	0.73	0.07	(0.59, 0.87)	<0.0001
*Total range* (*t1, t2*)	68%–82%	0.55–0.74			
*Mean* (*t1, t2*)	78%	0.69			
*Tear thickness* (*IEK*)					
Rater 1, rater 2, t1	66.2%	0.69	0.06	(0.57, 0.81)	<0.0001
Rater 1, rater 3, t1	67.7%	0.73	0.06	(0.61, 0.84)	<0.0001
Rater 2, rater 3, t1	72.3%	0.78	0.05	(0.68, 0.87)	<0.0001
Rater 1, rater 2, t2	75.4%	0.80	0.05	(0.71, 0.89)	<0.0001
Rater 1, rater 3, t2	69.2%	0.74	0.06	(0.68, 0.85)	<0.0001
Rater 2, rater 3, t2	78.5%	0.84	0.04	(0.77, 0.92)	<0.0001
*Total range (t1, t2)*	66%–79%	0.69–0.84			
*Mean (t1, t2)*	72%	0.76			
*AP extent* (*IEK*)					
Rater 1, rater 2, t1	61.5%	0.57	0.07	(0.43, 0.71)	<0.0001
Rater 1, rater 3, t1	61.5%	0.57	0.07	(0.42, 0.72)	<0.0001
Rater 2, rater 3, t1	78.5%	0.80	0.05	(0.70, 0.90)	<0.0001
Rater 1, rater 2, t2	78.5%	0.77	0.06	(0.65, 0.89)	<0.0001
Rater 1, rater 3, t2	64.6%	0.64	0.07	(0.51, 0.77)	<0.0001
Rater 2, rater 3, t2	75.4%	0.79	0.05	(0.70, 0.89)	<0.0001
*Total range (t1, t2)*	62%–79%	0.57–0.80			
*Mean (t1, t2)*	70%	0.69			
*Total score* (*IEK*)					
Rater 1, rater 2, t1	38.5%	0.65	0.05	(0.55, 0.75)	<0.0001
Rater 1, rater 3, t1	36.9%	0.63	0.05	(0.52, 0.73)	<0.0001
Rater 2, rater 3, t1	61.5%	0.80	0.04	(0.72, 0.89)	<0.0001
Rater 1, rater 2, t2	61.5%	0.80	0.04	(0.72, 0.88)	<0.0001
Rater 1, rater 3, t2	52.3%	0.71	0.05	(0.61, 0.81)	<0.0001
Rater 2, rater 3, t2	58.5%	0.80	0.04	(0.73, 0.88)	<0.0001
*Total range* (*t1, t2*)	39%–62%	0.63–0.80			
*Mean* (*t1, t2*)	52%	0.73			

The mean IEK for tendonosis was 0.69 (range: 0.55–0.74), for tear thickness 0.76 (range: 0.69–0.84), for AP tear size 0.69 (range: 0.57–0.80), and for total tendonopathy score 0.73 (range: 0.63–0.80). According to Landis and Koch [21], these mean kappa values reflect good agreement (0.61–0.80).

## Discussion

This study proposes a new MRI protocol for quantitative evaluation of supraspinatus tendonopathy. This protocol takes into account that tendonopathy can include partial thickness tears of varying extent which can occur in conjunction with focal or generalized tendonosis. Partial thickness cuff damage can be scored accordingly. The protocol refines assessment of the extent of partial tendon tearing, separately scoring tendon tear thickness (grades 1–4) and tear extent in the AP direction (grades 1–3). This MRI protocol therefore differentiates partial thickness tears to a greater extent compared to previous studies, which have looked at partial tears in the coronal plane only and evaluated as less than 50% or greater than 50% of tendon thickness [3,13]. A comprehensive three-dimensional evaluation is considered important for monitoring of disease progression with and without treatment. For example, a less than 50% partial thickness tendon tear on coronal MRI is generally considered low grade. However, when associated with significant extension in the AP dimension and with severe tendonosis, this partial tear will score more highly on the proposed MRI cuff scoring protocol and would be more at risk of disease progression compared to a low-grade partial thickness tear without tendonosis or extensive AP involvement.

This study finds intra-observer reliability good to excellent for each individual tendonopathy parameter and for the total score. With no prior familiarity with this MRI protocol and only a brief education session, each individual MSK radiologist applied this protocol to score the 65 MRI scans consistently.

Mean inter-observer reliability was good for all parameters and for the total tendonopathy score. Weighted kappa statistics indicated that assessment of AP extent of tendon tear was more variable than assessment of tendon tear thickness despite the latter involving two separate measurements and a calculation.

This study is purely a reliability study of 3 T MRI for morphological image grading of supraspinatus tendonosis and partial thickness tears and is most suitable as an objective imaging outcome measurement in the setting of clinical studies. We did not aim to assess clinical correlation since morphologically measurable changes of tendonopathy might well be subclinical; however, they enable the clinician to monitor the course of disease during follow-up.

Limitations of this study include the lack of arthroscopic correlation to validate the cuff pathology identified on the MRI scans. However, this study is a reliability study of a MRI scoring protocol and arthroscopic correlation is beyond the aims of this study. A further limitation is that only experienced MSK radiologists have evaluated the reliability of this MRI protocol and that 3 T MRI is not widely available as yet. Less experienced radiologists or non-radiology observers would most likely evaluate rotator cuff tendonopathy less reliably with this protocol. In addition, non-radiology observers would not have the large digital radiology monitors and InteleViewer measurement software available to the study observers. This MRI cuff protocol is likely to be less reliable in a general clinical setting.

The strengths of this study are the proposal of a new reliable MRI protocol for objective imaging outcome measurement of supraspinatus tendonosis and partial thickness tears. Such a system with reliability validation is lacking in many studies using MRI as an outcome measure. The protocol and its application are simple to learn and it has excellent intra-observer and good inter-observer reliability as shown in this study on a statistically robust number of tested MRI scans. This protocol will be a very useful tool for objective radiological outcome surveillance in the clinical setting but more so for research outcome measurement at single time points and during follow-up.

## Conclusion

A comprehensive MRI evaluation protocol for objective assessment of supraspinatus tendonopathy has been proposed. This protocol is simple to learn and has excellent intra-observer and good inter-observer reliability. This MRI protocol is designed to be used in surveillance of partial thickness cuff injuries and to evaluate the tendon response to treatment.

## Additional file

Additional file 1:
**MRI—scoring questionnaire.**
