# Supplementary material for: Reliability of a 3 T MRI protocol for objective grading of supraspinatus tendonosis and partial thickness tears
Source: J Orthop Surg Res. 2014 Dec 18;9:128. doi: 10.1186/s13018-014-0128-x (PMC4278262; doi:10.1186/s13018-014-0128-x)
Supplement: Additional file 1: — MRI—scoring questionnaire. [file 13018_2014_128_MOESM1_ESM.docx]

**Additional file 1**

MRI–scoring questionaire: Image number: Date of scoring: Number of scorer:

Please answer **all questions**

| *I Tendonosis* | Yes | | No |
| --- | --- | --- | --- |
| *PD* | | | |
| I.1 Is there a uniform low normal signal on pd weighted images? |  | |  |
| I.2 Measure the largest extent of signal increase on coronal pd: **mm** | | | |
| I.3 Measure the largest extent of signal increase on sagittal pd: **mm** | | | |
| I.4 Is there a focal (<10mm) signal increase on pd weighted images? | |  |  |
| I.5 Is there a generalized (>10mm) signal increase on pd weighted images? | |  |  |
| *T2* | | | |
| I.6 Is there a uniform normal tendon signal on t2 weighted images? | |  |  |
| I.7 Measure the largest extent of signal increase on coronal t2 (Fig 2) **mm** (not fluid signal!) | | | |
| I.8 Measure the largest extent of signal increase on sagittal t2 (Fig 2): **mm** (not fluid signal !) | | | |
| I.4 Is there a focal (<10mm) signal increase on t2 weighted images? | |  |  |
| I.5 Is there a generalized (>10mm) signal increase on t2 weighted images? | |  |  |
| *II Tear Thickness* | | | |
| II.1 Is there a partial thickness tear? | |  |  |
| II.2 Measure the largest tear thickness on coronal t2 perpendicular to the tendon fibres (fluid signal only): **mm** | | | |
| II.3 Determine the percentage tear to the normal tendon: **%** | | | |
| *III AP Tear Size* | | | |
| III.1 Is there a partial ap/partial thickness tear? | |  |  |
| III.2 Measure the largest ap tear size on sagittal t2: **mm** | | | |
